# Letter to the Editors: “Autopsy-based histopathological characterization of myocarditis after anti-SARS-CoV-2-vaccination” by C. Schwab et al.

**DOI:** 10.1007/s00392-023-02198-0

**Published:** 2023-04-25

**Authors:** Peter Schirmacher, Thomas Longerich, Constantin Schwab

**Affiliations:** Universitätsklinikum Heidelberg Pathologisches Institut, Heidelberg, Germany

Sirs,

We appreciate the interest of de Boer et al. in our autopsy study describing anti-SARS-CoV-2 vaccine-induced myocarditis. De Boer and colleagues argue that the performed investigations are incomplete and potential causes of death might have been missed, especially of toxicological, autoimmunological or genetic nature. Because, in some cases, even a full post-mortem examination might not identify a cause of death, they argue to not over-interpret “minor findings”, as this may prevent appropriate follow-up for diseases that are not identifiable at autopsy, such as genetic diseases.

We agree that pathologists are responsible for establishing the precise cause of death and that there is considerable variation in the way in which this complex task is approached. In particular, there are fundamental differences between clinical and forensic autopsy. And indeed, the guidelines for autopsy investigation of sudden cardiac death as published by the Association for European Cardiovascular Pathology are not fully met in terms of toxicology and genetic testing [1]. However, myocarditis due to SARS-CoV-2 mRNA vaccine is reported in numerous publications and as such is a proven fact [2–4]. Importantly, none of the patients reported in our study experienced a ‘death sine materia’, since the described histopathological changes cannot be considered as an unspecific finding and at least in the patients with presence of myocarditis are the likely cause of death [5]. Thus, additional toxicological or genetic testing—as a potential next analytical level—does not seem to be warranted in these patients. However, genetic testing might be able to identify factors that may predispose for an anti-SARS-CoV-2 vaccine-induced myocarditis, but the number of cases in our study is likely too small to allow for identifying a possible genetic association. As a consequence, a large international autopsy study would be required to finally answer such a scientific question, a task that has likely to be initiated by regulatory authorities.

As there is not a universally accepted definition of myocarditis, we applied the Dallas criteria that have been established for the diagnosis of myocarditis. The Dallas criteria were developed as endomyocardial biopsies are an important diagnostic tool in the clinical context and a reproducible scoring system is a prerequisite for therapeutic decision making. The main difference between endomyocardial biopsies and autopsy sections is the tissue area available for histological evaluation. Thus, the chance of detecting inflammatory foci is higher in autopsy specimen, thereby reducing the chance of a false-negative histology in the autopsy setting. This does not change the diagnostic concept of myocarditis. Myocyte destruction was defined by morphological features such as lack of myocytes at the site of an inflammatory foci or morphologically altered myocytes with condensed, eosinophilic cytoplasm and dense chromatin fragments at the margin of the nuclear membrane.

We postulate that acute arrhythmia (ventricular fibrillation or asystoly) was the cause of death, which is in line with the data by Corrado et al., who also reported that focal inflammatory infiltrates may precipitate fatal ventricular arrhythmias despite normal ventricular function and in the absence of prodromal symptoms [6]. Of note, Corrado et al. applied the Dallas criteria in their autopsy study, suggesting that they may provide relevant information in the autopsy setting as well. We assume that not the extent of the inflammatory infiltrate is of most importance but the localization (e.g., spatial relation to the conduction system) may matter. As already pointed out in our manuscript, a focused dissection and detailed histological evaluation of the cardiac conduction system was not part of our standardized autopsy procedure. Autopsy per se is unable to reliably identify the inflammatory focus, which has triggered the fatal course. However, cardiac arrest is an established serious complication of myocarditis; thus, the presence of myocarditis in context of the lack of autopsy findings suggesting chronic output failure or any other potential cause of death is a strong and generally accepted indication for cardiac arrest due to asystoly or ventricular fibrillation as the cause of death.

As stated in our paper, we did not see comparable CD4-dominated T cell infiltrates in 20 years of autopsy service—in which > 3000 autopsies were performed—and validated this impression by analyzing an age- and sex-matched control cohort as part of the reported study. We provide a clinical autopsy service; thus, histological evaluation is routinely performed as part of the standard workup at our institution.

Beyond any reasonable doubt and to the maximal extent possible for an autopsy study (which in other settings is also unequivocally accepted to establish the causality of disease for death, such as profession-associated death and late death due to accident), our study has established the morphological characteristics of anti-SARS-CoV-2 vaccine-induced (epi-)myocarditis, and its causality for death. The different arguments have already been substantiated in our publication, i.e., exclusion of any other (especially infectious) cause and absence of any other heart disease, time context, consistent morphology and immunology suggestive of an immune-mediated myocarditis. Co-existence of phenotypically identical inflammatory infiltrates at the deltoid jab site cannot be sufficiently explained by a vaccine-unrelated, autoimmune or genetic cardiac disease. We fully agree that more research is required to characterize the pathomechanisms of COVID-19 vaccine-related unwanted effects but its existence and phenotype is a fact.

## Data Availability

As for the original paper data are available upon reasonable request.
